# Dietary Supplementation with *Enterococcus faecium* R1 Attenuates Intestinal and Liver Injury in Piglets Challenged by Lipopolysaccharide

**DOI:** 10.3390/ani11051424

**Published:** 2021-05-16

**Authors:** Yanyan Zhang, Tao Wu, Zhenqiang Chen, Yuxuan Meng, Zhekun Zhu, Qian Wang, Junjie Tian, Dan Yi, Lei Wang, Di Zhao, Yongqing Hou

**Affiliations:** Hubei Key Laboratory of Animal Nutrition and Feed Science, Wuhan Polytechnic University, Wuhan 430023, China; zhangyanyana305@163.com (Y.Z.); wutao@whpu.edu.cn (T.W.); chen07928263206@163.com (Z.C.); m13871101699@163.com (Y.M.); zhuzhekun1207@163.com (Z.Z.); w1353807950@163.com (Q.W.); tianjunjie9523@163.com (J.T.); yidan810204@whpu.edu.cn (D.Y.); wanglei_wh@aliyun.com (L.W.); zhaodi@whpu.edu.cn (D.Z.)

**Keywords:** weaned piglet, growth performance, *E. faecium* R1, lipopolysaccharide, intestine, liver

## Abstract

**Simple Summary:**

The biological characteristics of *E. faecium* R1 and the effect of dietary supplementation with *E. faecium* R1 on the growth performance of weaned piglets were studied. The results showed that *E. faecium* R1 had the characteristics of effective bacteriostatic activity, acid resistance, bile salt resistance, and high-temperature resistance. Dietary supplementation with *E. faecium* R1 (6.5 × 10^6^ CFU/g) improved intestinal function of weaning piglets by decreasing diarrhea incidence. Further research found that dietary supplementation with *E. faecium* R1 (6.5 × 10^6^ CFU/g) attenuated intestinal and liver injury in piglets challenged by lipopolysaccharide.

**Abstract:**

In this study, a strain of *E. faecium* R1 with effective bacteriostatic activity, acid resistance, bile salt resistance, high-temperature resistance was screened. To study the effect of *E. faecium* R1 on lipopolysaccharide (LPS)-induced intestinal and liver injury in piglets, twenty-four weaned female piglets were randomly assigned into one of three groups (8 piglets per group). Piglets in the control group and LPS group were fed a basal diet, piglets in the *E. faecium* group were fed the basal diet supplemented with *E. faecium* R1 (6.5 × 10^6^ CFU/g). On day 21 of the trial, piglets in the LPS group and *E. faecium* group were intraperitoneally administered LPS (100 μg/kg), piglets in the control group were administered the same volume of saline. Subsequently, blood samples were collected at 3 h, and intestinal, liver, and pancreas samples were collected at 6 h. Results showed that *E. faecium* R1 supplementation significantly decreased the diarrhea rate and feed to gain ratio, and dramatically reduced LPS-induced intestinal and liver injury in piglets. Compared with the LPS group, *E. faecium* R1 supplementation significantly increased the content of glucagon in plasma and IL-1β in the liver, and the mRNA levels of *villin* in jejunum and ileum and *Bcl-xL* and *pBD-L* in the ileum, and significantly decreased the contents of prostaglandin 2 and malondialdehyde in the liver and the activities of myeloperoxidase and aspartate aminotransferase in plasma in piglets. Moreover, *E. faecium* R1 improved the pancreatic antioxidant capacity in piglets, which was indicated by a significant increase in catalase activity and a decrease in total nitric oxide synthase activity. In summary, dietary supplementation with *E. faecium* R1 alleviates intestinal and liver injury in LPS-challenged piglets.

## 1. Introduction

Weaning and the weaning diet seriously threaten the intestinal health of piglets. Diarrhea caused by weaning and the weaning diet is a very common disease, which eventually develops into growth retardation and seriously restricts the development of the pig industry [1,2,3]. Therefore, the feeding of weaned piglets directly affects the growth of pigs during the fattening period [4,5]. Although feed antibiotics can effectively alleviate diarrhea and reduce the incidence of disease in piglets, many countries have banned the use of in-feed antibiotics because of their potential harm to animal production [6,7]. Therefore, healthy and safe antibiotic substitutes will be very important for the healthy growth of animals. Many studies have shown that probiotics can prevent diarrhea in piglets, enhance immune function and improve the growth performance of piglets [8,9,10,11,12]. Probiotic preparation is a kind of micro-ecological preparation made from living microorganisms, which have been widely used in animal farming in recent years and to improve the growth performances of animals [9].

*Enterococcus faecium* (*E. faecium*) is a gram-positive coccus and belongs to the *Enterococcus* genus. In addition, *E. faecium* is a kind of facultative anaerobic lactobacillus that can colonize the digestive tract of humans and animals [10]. Some studies have reported that *Enterococcus* can promote the growth of animals by forming dominant microorganisms in the gastrointestinal tract [11,12,13,14]. Therefore, *E. faecium* may be used as a potential antibiotic substitute in animal production to improve the safety of livestock and poultry products.

Although probiotics are safe and effective antibiotic substitute, not all probiotics can be used as feed additives due to the complex physiological environment of the gastrointestinal tract. Because most microorganisms that enter the stomach cannot survive in the acidic gastric environment [15]. Even if a few microorganisms survive in the stomach, they have to resist a strong destructive effect of bile salts in the small intestine on the cell membrane [16,17,18]. Besides, high temperature during feed pelleting can kill most probiotics [19,20]. In short, stomach acid, bile salts, and high temperature seriously affect the application value of probiotics in feed. Therefore, to produce safe and efficient probiotic preparations, the screened probiotic strains can not only promote animal growth but also have stable biological characteristics.

In this study, a strain of *E. faecium* R1 with stable biological characteristics was screened from multiple probiotic strains. We hypothesized that dietary supplementation with *E. faecium* R1 may improve the growth performance of weaned piglets and alleviate tissue damage in piglets challenged by LPS. The purpose of this research was to test this hypothesis and to elucidate the underlying molecular mechanisms. 

## 2. Materials and Methods

### 2.1. Strains and Growth Condition

Ten strains of probiotic were isolated from intestinal contents in pigs fed commercial compound feeds according to the general method described previously [21,22], which were grown at 37 °C in MRS medium. *Escherichia coli* K88 *(E. coli* K88) was gifted by the key laboratory of agricultural microbiology, Huazhong Agricultural University, which was cultured in LB medium.

### 2.2. Biological Characteristics of Isolated Probiotics

To determine the bacteriostasis of isolated probiotic strains against pathogenic *E. coli* K88, the bacteriostasis circle diameters were determined by the oxford cup method [23] with minor modifications. First of all, the pathogenic *E. coli* K88 was cultured for 12–24 h and diluted 20 times. Secondly, 100 µL of *E. coli* K88 diluent was evenly plated on the sterile agar plate. Finally, the oxford cup was placed vertically on the surface of the culture medium and gently press to make it contact with the culture medium without any gaps. Subsequently, the probiotic strains (100 µL/cup, 1.0 × 10^7^ CFU/mL) were added to the oxford cup and cultivated at 37 °C for 1–18 h. The bacteriostasis circle diameters were measured by using vernier calipers.

To determine the tolerance of isolated probiotic strains to high temperature, these probiotic strains cultured to logarithmic phase were transferred to sterile phosphate-buffered saline (PBS) (1:100). Before incubation in a water bath, the number of initial probiotic strains in PBS was counted by plate counting method, respectively. After being incubated in PBS respectively in a water bath at 60 °C, 75 °C, or 90 °C for 10 min, the number of probiotic strains was counted by plate counting method. Finally, survival rates were calculated separately. Survival rate (%) = the number of probiotic strain after culture for 10 min at 60 °C, 75 °C, or 90 °C/the amount of initial probiotic strain × 100.

To determine the tolerance of isolated probiotic strains to acid, these probiotic strains cultured to logarithmic phase were transferred to PBS (1:100) with different pH values (1.5, 2.5, 3.5), respectively. After incubation at 37 °C for 3 h, the number of the probiotic strains in PBS with different pH was separately counted by plate counting method. Finally, survival rates were calculated separately. Survival rate (%) = the number of probiotic strains after culture for 3h at different pH values (1.5, 2.5, 3.5)/the amount of initial probiotic strain × 100. 

To determine the tolerance of isolated probiotic strains to bile salt, these probiotic strains cultured to logarithmic phase were transferred to PBS (1:100) with different concentrations of bile salts (0.1%, 0.3%, and 0.5%), respectively. After incubation at 37 °C for 3 h, the number of the probiotic strains in PBS with different concentrations of bile salts was separately counted by the plate counting method. Finally, survival rates were calculated separately. Survival rate (%) = the number of probiotic strain after culture for 3h at different concentrations of bile salts (0.1%, 0.3%, and 0.5%) / the amount of initial probiotic strain × 100.

### 2.3. Animals and Experimental Design

The study was conducted after approval by the Institutional Animal Care and Use Committee at Wuhan Polytechnic University (No. WPU202011002). 

To study the effect of *E. faecium* R1 on the growth performance of weaned piglets, twenty-four 3-week-old healthy crossbred female piglets (Duroc × Landrace × Yorkshire) were weaned and purchased from a commercial pig farm. The feeding and management of animals were performed as previously described [24] with some modifications. After a 4-day adaptation period, piglets (25 days of age, average body weight of 6.50 ± 0.43 kg) were randomly assigned into one of three groups (8 piglets per group). Group 1, which was fed the basal diet, served as a control group. Group 2, which was fed the basal diet with subsequent lipopolysaccharide (LPS) challenge on day 21, served as the LPS group. Group 3, which was fed the basal diet supplemented with *E. faecium* R1 (6.5 × 10^6^ CFU/g), served as *E. faecium* group. The three groups were housed individually in stainless steel metabolic cages (1.20 × 1.10 m^2^) and maintained in an environmentally controlled room (25 °C) by air conditioning. During a 4-day adaptation period, all piglets had free access to the basal diet to help them adapt to solid food. The day on which the basal diet supplemented with *E. faecium* R1 was fed was designated as day 1 (25 days of age). *E. faecium* R1 (powder) which was prepared by the freeze-drying method was thoroughly mixed with basal diet (the final concentration of 6.5 × 10^6^ CFU/g *E. faecium* R1) before each feeding. The dosage of 6.5 × 10^6^ CFU/g *E. faecium* R1 was chosen because we found that dietary supplementation with *E. faecium* R1 (≥6.5 × 10^6^ CFU/g) could improve the growth performance of piglets in pre-experiment. The corn and soybean meal-based diet (Table 1) was formulated to meet National Research Council’s (NRC, 2012) recommended requirements for all nutrients [24]. The content of calcium (Ca), total phosphorus (P), and crude protein (CP) in dietary was analyzed according to the Weende method of the feed proximate analysis as described by Henneberg and Stohmann [24]. The content of tryptophan, cystine, methionine, threonine, and total lysine in diets was analyzed by an automatic amino acids analyzer (S433D, Sykam GmbH, Eresing, Germany) [24].

During days 1–21 of the trial prior to the LPS challenge, the body weight, feed intake, and diarrhea of piglets were recorded to statistically analyze their growth performance. Piglets were weighed once a week and the feed intake was recorded daily. To determine the diarrhea rate, individual pigs were examined for diarrhea four times per day. Diarrhea was quantified by the following equation for each pig. Diarrhea rate (%) = total diarrhea incidences/8 × 100. Average daily gain (Kg/day) = gain (Kg)/21. Average daily fed intake (Kg/day) = total feed intake (Kg)/21. Feed/gain ratio (%) = total feed intake (Kg)/gain (Kg) × 100.

On day 21, the LPS group and *E. faecium* group were injected intraperitoneally with LPS (100 μg/kg bodyweight) (*Escherichia coli* serotype O55: B5; Sigma) dissolved in sterile saline. The control group was injected with the same volume of sterile saline. At 3 h post LPS challenge, blood samples were collected from the anterior vena cava into heparinized vacuum tubes and centrifuged at 1000 × g for 10 min at 4 °C, and then the separated blood plasma was divided into sterile tubes and stored at −80 °C for further analysis. At 6 h post LPS challenge, all piglets were euthanized by deep anesthesia with pentobarbital sodium (50 mg/kg bodyweight). Subsequently, the liver and pancreas were collected from the pig abdomen and then immediately stored at −80 °C until assay. Four 10 cm intestinal segments were respectively cut off from duodenum, jejunum, ileum, and colon and then gently flushed with ice-cold 0.9% saline. Subsequently, intestinal mucosae were scraped off from the intestinal segments and immediately frozen in liquid nitrogen. All samples were collected within 15 min after killing and stored at −80 °C for further analysis.

### 2.4. Biochemical Indices in Plasma and Liver

The blood plasma was taken out from the −80 °C ultra-low temperature refrigerator and placed at 4 °C until it melts. The liver tissue (~200 mg) was homogenized in a nine-fold volume of freezing saline and then centrifuged at 2500 rpm for 10 min at 4 °C to obtain the supernatant fluid used for assays. Insulin (INS), cortisol (COR), glucagon (GC), prostaglandin 2 (PGE2), interleukin-1β (IL-1β), insulin-like growth factor 1 (IGF-1) in the plasma, and IGF-1, PGE2, and IL-1β in the liver were determined using commercially available PGE2 (HY-10047) RIA kit purchased from Beijing Huaying Institute of Biology and Insulin (KAP1251) assay kit, COR (KAPDB280) assay kit, GC (RV07-152101) assay kit, IL-1β (KAP1211) assay kit and IGF-1 (KAP1581) assay kit purchased from Beijing North Institute of Biology [24,25]. The assays were performed in triplicate.

### 2.5. Determination of Antioxidant Capacity and Oxidation-Related Product

Plasma and liver samples were analyzed for the activities of ant-oxidative enzymes and related products. Catalase (CAT), myeloperoxidase (MPO), total nitric oxide synthase (tNOS), inducible nitric oxide synthase (iNOS), glutathione peroxidase (GSH-Px), and malondialdehyde (MDA) were determined using commercially available catalase (A007-2-1) assay kit (Ultraviolet), myeloperoxidase (A044-1-1)assay kit, nitric oxide synthase (NOS) typed assay kit (Colorimetric method), glutathione peroxidase (GSH-PX) assay kit and malondialdehyde (MDA) assay kit (TBA method) purchased from Nanjing Jiancheng Bioengineering Institute [24,26]. The assays were performed in triplicate.

### 2.6. Determination of Intestinal Absorption Function and Disaccharidase Activity

The activities of intestinal disaccharidases (lactase, sucrase, and maltase) determined using glucose kits (Cat. A082-1, A082-2, and A082-3 for lactase, sucrase, and maltase, respectively), were analyzed as previously described [27]. D-Xylose in plasma was determined as described by Yi et al. [27]. The concentration of D-xylose in plasma were determined using commercially available kits (Cat. A035-1-1) purchased from Nanjing Jiancheng Bioengineering Institute. The assays were performed in triplicate.

### 2.7. Quantitative PCR (qPCR) for Gene Expression Analyses

Total RNA was isolated from approximately 100 mg of each frozen intestinal sample as previously described [25]. The qualified RNA was reverse-transcribed to cDNA for qPCR as previously described [26]. The primers [27] were designed according to the genomic sequence of the pig. Ribosomal protein L4 (RPL4) and glyceraldehyde-3-phosphate dehydrogenase (GADPH) were used as internal control genes. Each biological sample was run in triplicate.

## 3. Statistical Analysis

Data are expressed as means ± SD and analyzed by one-way analysis ANOVA in the SPSS 16.0 software (Chicago, IL, USA). Differences among treatment means were determined by Duncan’s multiple comparison test. Additionally, the difference in the means of growth performance was determined by the Student’s paired *t*-test. *P* < 0.05 was taken to indicate statistical significance [26,27].

## 4. Results

### 4.1. Biological Characteristics of E. faecium R1

In this study, ten strains of probiotics (*E. faecium* R 1, *E. faecium* R2, *Bacillus subtilis* (*B. subts*), *Lactobacillus casei* Q (*L. casei* Q), *L. casei* G, Probiotic G-Q, Probiotic G-G, *Lactobacillus Bacillus* (*L. Bacillus*), *Lactobacillus acidophilus* (*L. acidophilus*), and *Enterococcus lactis* (*E. lactis*)) were isolated and identified successfully. *E. faecium* R 1, *E. faecium* R2, and *E. lactis* belong to Enterococcus genus. *B. Subts* belong to *Bacillus genus.* *L. casei* Q, *L. casei* G, *L. acidophilus* and *L. Bacillus* belong to the *Lactobacillus genus*. Probiotic G-Q and Probiotic G-G belong to *Bifidobacterium genus*. The inhibitory effects of ten probiotic strains to E. coli K88 in vitro are shown in Table 2. These results showed that all ten probiotic strains have bacteriostatic properties against *E. coli* K88, of which *E. lactis*, *E. faecium* R1, *E. faecium* R2, and Probiotic G-G exhibited excellent antibacterial activity against *E. coli* K88. The diameters of the inhibition zone were 18.21, 17.94, 17.53, and 17.34 mm, respectively.

**Figure 1:**
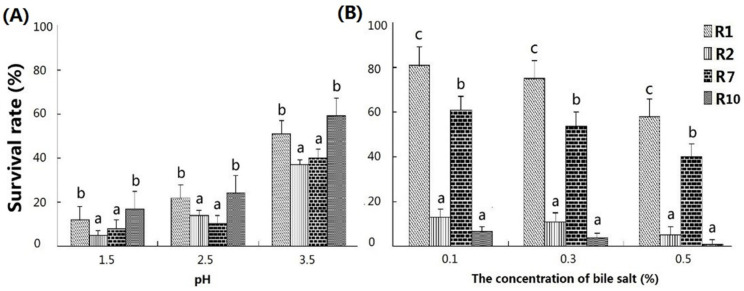
Analysis of acid and bile salt tolerances of 4 probiotic strains (R1, R2, R7, and R10). (**A**) Survival rates of R1, R2, R7 and R10 in acid (pH = 1.5, 2.5 or 3.5) condition, respectively; (**B**) Survival rates of R1, R2, R7 and R10 in 0.1%, 0.3% and 0.5% bile salt medium, respectively. R1 = *E. faecium* R1; R2 = *E. faecium* R2; R7 = *Probiotic* G-G; R10 = *E. lactis*. a,b,c Within a group, means with different superscripts differ (*p* < 0.05).

Subsequently, the tolerances of *E. lactis*, *E. faecium* R1, *E. faecium* R2, and Probiotic G-G to acid, bile salt, and the high temperature were tested, respectively. The acid tolerances of these four strains are shown in Figure 1A. After incubation in an acidic environment (pH = 1.5, 2.5, or 3.5) for 3 h at 37 °C, the survival rates of *E. faecium* R1 and *E. lactis* were significantly higher than those of *E. faecium* R2 and Probiotic G-G. The tolerances of these four strains to bile salt showed a great difference (Figure 1B). After incubation in PBS with 0.1, 0.3 or 0.5% bile salt for 3 h at 37 °C, the survival rates of *E. faecium* R1 and *E. lactis* were significantly higher than those of *E. faecium* R2 and Probiotic G-G, and the survival rate of *E. faecium* R1 was significantly higher than that of *Probiotic* G-G. After incubation in a water bath at 60 °C, 75 °C or 90 °C for 10 min, the survival rates of all these four strains were less than 1% at 75 °C and 90 °C (data not shown). When the temperature was 60 °C, the survival rate of *E. faecium* R1 (86.78%) was the highest among these four strains and the number of viable *E. faecium* R1 (7.81 × 10^7^ CFU/mL) was significantly more than those of *E. faecium* R2 (1.52 × 10^7^ CFU/mL), *Probiotic* G-G (7.81 × 10^7^ CFU/mL), and *E. lactis* (3.50 × 10^7^ CFU/mL) (Table 2).

### 4.2. The Growth Performance of Piglets

Prior to the LPS challenge, no significant difference in average daily gain (ADG) of piglets in the *E. faecium* group compared with that in the control group was found. During days 15–21, the feed to gain (F/G) ratio of piglets in the *E. faecium* group was significantly decreased compared with that in the control group. During days 0–21, the diarrhea rate (DR) of piglets in the *E. faecium* group was significantly lower than that in the control group. DR of piglets in the *E. faecium* group decreased by 45.96% compared with that in the control group (Table 3).

### 4.3. Plasma Biochemical Parameters

Thirteen plasma biochemical indicators are determined and shown in Table 4. After the LPS challenge, plasma AST activity and the concentration of plasma TBIL in the piglets in the LPS group were significantly increased compared with those in the control group. In contrast, plasma AST activity in piglets in the *E. faecium* group was significantly decreased compared with that in the LPS group. No significant difference was found in the plasma AST activity in piglets between the *E. faecium* group and the control group. However, the concentration of plasma TBIL in the piglets in the *E. faecium* group did not differ from that in the LPS group. No significant differences were found in other indices.

### 4.4. The Concentrations of Inflammatory Mediators and Hormones in Plasma

Six plasma and three liver indices of inflammatory mediators and hormones are determined and shown in Table 5. After the LPS challenge, the concentrations of COR and IGF-1 in the plasma of piglets in the LPS group were significantly higher than those in the control group. The concentrations of plasma COR and liver IL-1β in the piglets in the *E. faecium* group were significantly higher than those in the LPS group. The concentration of plasma GC in the piglets in the *E. faecium* group was significantly higher than that in the control group. The concentration of PGE2 in the liver of piglets in the *E. faecium* group was significantly decreased compared with that in the LPS group. No significant differences were found in other indices.

### 4.5. Anti-Oxidative Enzymes and Oxidation Products in the Plasma, Liver, and Pancreas

The parameters which are related to anti-oxidative enzymes and oxidation products in this study are shown in Table 6. After the LPS challenge, CAT activity in the pancreas of piglets in the LPS group was significantly decreased compared with that in the control group. Plasma MPO activity in the piglets in the LPS group was significantly increased compared with that in the control group. Dietary supplementation with *E. faecium* R1 significantly increased CAT activity in the pancreas, and significantly decreased total nitric oxide synthase (tNOS) activity in the pancreas and the content of MDA in the liver of piglets in *E. faecium* group compared with that in the LPS group.

### 4.6. Effects of E. faecium R1 on Intestinal Function in the Piglets Challenged with LPS

Indicators related to intestinal absorption function in piglets are shown in Table 7. The contents of plasma D-Xylose and alkaline phosphatase (AKP), lactase, and sucrase were significantly decreased compared with those in the control group. However, compared with the LPS group, dietary supplementation with *E. faecium* R1 tended to increase (*p* < 0.1) the contents of lactase and sucrase in the jejunum of piglets.

### 4.7. The mRNA Levels of Villin, Bal-xL, and pBD-1 in the Small Intestine

The mRNA levels of *villin* in jejunum and ileum in piglets in the LPS group were significantly decreased compared with that in the control group. Compared with the LPS group, dietary supplementation with *E. faecium* R1 significantly increased the mRNA levels of *villin* in the jejunum and ileum in piglets. Meanwhile, the mRNA levels of *pBD-1* and *Bal-xL* in the ileum of piglets in the *E. faecium* group were significantly increased compared with those in the LPS group (Table 8).

## 5. Discussion

After weaning, the changes of diet from the sow milk to solid food often weaken the ability of piglets to adapt to the environment. Weaned piglets are easily infected by pathogenic microorganisms. The proliferation of pathogenic microorganisms in the host is usually accompanied by reduced feed intake, activation of the immune system, and growth retardation [28,29,30]. Therefore, post-weaning diarrhea is a very common disease that seriously threatens the healthy growth of weaned piglets [31,32,33]. In recent years, probiotics have been used as feed additives with an in-depth study of animal microecology [34,35].

Although many probiotics are currently used as feed additives, the actual effects of microecological agents prepared by different probiotics are different in animals [36,37]. Some studies have reported that probiotics must adhere to the intestinal epithelium cells and colonize in animal intestines to function [11,12,13,14]. However, the high temperature produced during feed pelleting, the acidic environment of the stomach, and high concentrations of bile salts in the animal gastrointestinal tract can kill the vast majority of microorganisms [15,16,17,18,19]. Therefore, probiotics that can be used to prepare microecological agents must have the biological characteristics of acid, salt, and high-temperature resistance.

Some studies have shown that probiotics can promote the healthy growth of animals by the formation of dominant microflora to inhibit the survival of pathogens and promote nutrient absorption in the intestine [4,38]. Therefore, probiotics used to prepare microecological agents must have the ability to regulate intestinal flora and enhance intestinal barrier function. In this study, the bacteriostatic effects of 10 probiotic strains isolated from intestinal contents in pigs fed commercial compound feeds were determined. Four probiotic strains (*E. lactis*, *E. faecium* R1, *E. faecium* R2, and *Probiotic* G-G) showed a highly effective bacteriostasis against pathogenic *E. coli* K88 compared with other strains in vitro. In addition, probiotics used as feed additives not only possess effective antimicrobial effects but also need to be successfully colonized in animal intestines. Since the pH in the pig’s stomach is 2–3.5, probiotics with acid resistance can survive in a pig’s stomach and enter the intestine. Once probiotics enter the duodenum, they will encounter a high concentration of bile salts (0.03–0.3%) secreted by the liver, which possesses bacteriostatic activity and can destroy the cell membrane by causing cell membrane rupture and membrane protein dissociation [22,23]. In addition to the two aspects mentioned above, the high temperature (90 °C) produced during feed pelleting will cause the death of most probiotics. Therefore, before preparing probiotic preparations, the biological characteristics of selected probiotics need to be determined. The results of this study showed that *E. faecium* R1 and *E. lactis* not only had effective bacteriostatic properties but also had relatively higher survival rates than the other two strains (*E. faecium* R2 and *Probiotic* G-G) under acidic condition (pH 2.5). Moreover, *E. faecium* R1 was much more tolerant to pig bile salt solution than *E. lactis*. *E. faecium* R1 had the strongest heat resistance among the four strains at 60 °C, and its survival rate was more than 80%. Therefore, *E. faecium* R1 can be added to pig feed as a candidate probiotic strain because of its stable biological characteristics.

Previous studies have shown that *E. faecium* can adhere to the intestinal epithelium cells and colonize in the human and animal digestive tract, and secrete various lactic acid, amino acids, and vitamins in the process of metabolism, and promote the digestion and absorption of nutrients [10,39,40]. Taras et al. reported that although dietary supplementation with *E. faecium* reduced the number of pathogens in the intestine of weaned piglets and lowered the diarrhea rate, no significant difference was found [41]. However, our results showed that dietary supplementation with *E. faecium* R1 significantly lowered the diarrhea rate in weaned piglets. Strompfová et al. reported that dietary supplementation with *E. faecium* EK13 did not significantly improve feed conversion and growth performance of weaned piglets [42]. However, this study showed that dietary supplementation with *E. faecium* R1 significantly increased the feed conversion rate of weaned piglets after feeding for 15 days. The daily gain of weaned piglets in this study has also been increased. These results indicated that dietary supplementation with *E. faecium* R1 improved the growth performance of weaned piglets.

Although our results showed that dietary supplementation with *E. faecium* R1 can significantly improve the growth performance of weaned piglets, the underlying mechanism is still unknown. The changes in blood biochemical indexes in pigs are a reflection of changes in cell permeability and metabolic function of animals [43]. Some studies have shown that increased activity of alanine aminotransferase (ALT), aspartate aminotransferase (AST), and alkaline phosphatase (ALP) in the blood is usually a marker of the stress response and also indicates liver damage [44,45]. Consistent with previous studies indicating that LPS could induce the elevation of liver damage-related indicators in blood [24,26], the results of this study showed that the activity of AST in the blood of piglets was significantly increased after the LPS challenge. However, dietary supplementation with *E. faecium* R1 significantly decreased the activity of AST in the blood. The above results revealed that dietary supplementation with *E. faecium* R1 can attenuate liver injury in LPS-challenged piglets. Glucagon (GC) is a hormone secreted by islet B cells to increase blood sugar concentration. When the body is under intense stress, the secretion of glucagon is increased significantly, which promotes the rise of blood sugar and provides energy for the body to fight against stress [46]. The results of this study showed that dietary supplementation with *E. faecium* R1 promoted the secretion of glucagon when piglets were challenged with LPS. Previous studies have shown that cortisol (COR) is a hormone produced in response to stress [47]. After the LPS challenge, the contents of COR in the blood of piglets fed with or without dietary supplementation of *E. faecium* R1 were increased, which showed that *E. faecium* R1 cannot inhibit the stress response. These results revealed that *E. faecium* R1 can help piglets cope with stress response by effectively increasing the concentration of blood glucagon. Previous studies have reported that prostaglandin (PGE2) is a kind of inflammatory mediator that can cause a typical inflammation reaction [48]. The results of this study showed that dietary supplementation with *E. faecium* R1 significantly decreased the content of PGE2 in the liver of piglets challenged with LPS. 

Oxidative stress refers to the excessive production of free radicals such as oxygen free radicals (ROS) and reactive nitrogen free radicals (RNS), which can cause cytotoxic effects. If the antioxidants in the body can’t remove the oxides timely, the balance between the oxidant production system and the antioxidant system is broken, then the oxidative injury will occur in the body [49]. Normally, the body can scavenge excessive free radicals by antioxidant enzymes and antioxidants to protect the body from oxidative damage [24,49]. Enzymes of the antioxidant defense system mainly include SOD, CAT, GSH, GSH-Px (glutathione peroxidase) [50]. Catalase (CAT) is an enzyme that catalyzes the decomposition of hydrogen peroxide into oxygen and water, alleviates oxidative stress, and improves the antioxidant capacity of the body [51]. The result of this study showed that dietary supplementation with *E. faecium* R1 significantly increased the activity of CAT in the pancreas. Previous studies have shown that tNOS is activated to produce a large amount of NO which easily binds to O^2−^ to form ONOO^−^ to induce lipid peroxidation injury [52]. The result of this study showed that dietary supplementation with *E. faecium* R1 significantly decreased the activity of tNOS in the pancreas. As for malondialdehyde (MDA), a cytotoxic oxidation product, which can be significantly increased under the body’s oxidative stress [4,49]. Dietary supplementation with *E. faecium* R1 significantly reduced the content of MDA in the liver. These results revealed that dietary supplementation with *E. faecium* R1 can improve the antioxidant capacity of piglets by significantly increasing CAT activity in the pancreas, and decreased tNOS activity in the pancreas and MDA content in the liver.

The small intestine is the primary digestion and absorption organ of nutrients. When the morphology and function of intestinal in piglets are damaged, the piglet growth will be affected. D-xylose is a pentacarbose that can be absorbed by the small intestine without digestion and is generally not utilized by animals [53]. The determination of D-xylose in the blood can indirectly reflect the intestinal structure and function of piglets. Alkaline phosphatase (AKP) is a membrane-binding enzyme widely distributed in animal liver, bone, intestinal mucosa, and other tissues; it can decompose phosphate bonds in an alkaline environment [4]. Studies have reported that AKP plays an important role in the metabolic process of animals and serum AKP is closely related to the absorption and transportation of proteins, fats, and sugars [4,54]. Meanwhile, AKP in intestinal mucosal epithelial cells is a marker enzyme, which is closely related to intestinal digestion and absorption and can promote the absorption of amino acids, lipids, glucose, vitamin D, calcium, and phosphorus in the intestine [55,56]. Other studies reported that AKP has an important role in the clearance and detoxification of endotoxin, such as lipopolysaccharide produced by harmful microorganisms in the intestinal tract, and the protection of intestinal health [56]. Carbohydrates usually account for 50% to 80% of animal diets, and all carbohydrates are absorbed only after they are hydrolyzed into monosaccharides in the small intestine [49,57]. Intestinal disaccharidases are the key enzymes for carbohydrate digestion and absorption. The activity of disaccharidase can be used as a measurement of the development and function of intestinal mucosal epithelial cells [58]. Polysaccharides and oligosaccharides must be firstly degraded to disaccharides by digestive enzymes, and then disaccharides are decomposed into monosaccharides by disaccharidase before they can be absorbed [27,49,59]. In this study, LPS challenge significantly reduced the contents of D-xylose, AKP, disaccharidases, revealing that LPS challenge can significantly decrease the absorption function of the small intestine in piglets. However, dietary supplementation with *E. faecium* R1 did not significantly alter the content of D-xylose, AKP, disaccharidases, indicating that *E. faecium* R1 did not significantly improve intestinal absorption function in piglets. Moreover, the results showed that dietary supplementation with *E. faecium* R1 improved enterocyte proliferation in piglets, which is indicated by the significant increase of the gene expression of *villin* in jejunum and ileum and anti-apoptotic *Bcl-xL* in the ileum. Meanwhile, compared with the LPS group, dietary supplementation with *E. faecium* R1 significantly up-regulated the expression of the *pBD-1* gene in the ileum of piglets. pBD-1 is a widely distributed defensin in pigs and has broad-spectrum antimicrobial activity.

## 6. Conclusions

In summary, *E. faecium* R1 can be used as a safe and effective alternative to antibiotics because of the biological characteristics of acid, salt, and high-temperature resistance, as well as dietary supplementation with *E. faecium* R1 not only improves the intestinal function of piglets by decreasing diarrhea incidence but also alleviates intestinal and liver injury in piglets challenged by LPS. 

## Figures and Tables

**Table 1:** Ingredients and contents of energy and nutrients of the basal diet.

Ingredients	Content (%)
Corn (DE 14.27 MJ/kg, CP 8.7%)	61.88
Soybean meal l (DE 13.5 MJ/kg, CP 40%)	21.98
Wheat Middling (DE 13.4 MJ/kg, CP 13%)	4.00
Dried whey (CP 12%)	3.00
Fish meal (CP 66%)	3.00
Soy protein concentrate (CP 65%)	1.50
CaHPO 4	1.25
Premix ^†^	1.00
Limestone (CaCO 3 > 35%)	0.69
NaCl	0.30
Acidifier (Citric acid > 99%)	0.30
Soybean oil	0.50
L-Lysine HCl (78.8% lysine)	0.25
Choline chloride	0.20
Mold inhibitor (Calcium propionate > 30%)	0.10
DL-Methionine (99% methionine	0.05
Nutrients composition	
Digestible energy ^‡^ (MJ/kg)	14.22
Crude protein (%) ^§^	20.9
Total threonine (%) ^§^	0.74
Total methionine (%) ^§^	0.30
Total lysine (%) ^§^	1.15
Total tryptophan (%) ^§^	0.21
Total calcium (%) ^§^	0.70
Total phosphorus (%) ^§^	0.60
Available phosphorus (%) ^‡^	0.32

^†^ Premix provided the following amounts of vitamins and trace minerals per kilogram of the complete diet: ferrum, 100 mg (FeSO_4_·H_2_O); copper, 150 mg (CuSO_4_·5H_2_O); manganese, 40 mg (MnSO_4_·5H_2_O); zinc, 100 mg (ZnSO_4_·7H_2_O); iodine, 0.5 mg (KI); selenium, 0.3 mg (Na_2_SeO_3_·5H_2_O); vitamin A acetate, 3.66 mg; cholecalciferol, 0.10 mg; DL-α-tocopheryl acetate, 36.4 mg; menadione, 4 mg; thiamin, 6 mg; riboflavin, 12 mg; pyridoxine, 6 mg; cyanocobalamin, 0.05 mg; biotin, 0.2 mg; folic acid, 2 mg; niacin, 50 mg; D-calcium pantothenate, 25 mg; ^‡^ Calculated value; ^§^ Analyzed value.

**Table 2:** Analysis of bacteriostasis and heat resistance of probiotics.

Strains	Diameters of the Inhibition Zone (mm)	Survival Rate (60 °C)
R1	17.94	86.78
R2	17.53	17.88
R3	14.32	
R4	16.68	
R5	16.12	
R6	15.98	
R7	17.34	63.01
R8	16.21	
R9	16.52	
R10	18.21	19.77

R1 = *E. faecium* R1; R2 = *E. faecium* R2; R3 = *B. subtilis*; R4 = *L. casei* Q; R5 = *L. casei* G; R6 = Probiotic G-Q; R7 = Probiotic G-G; R8 = *L. Bacillus*; R9 = *L. acidophilus*; R10 = *E. lactis*.

**Table 3:** Effects of dietary supplementation with *E. faecium* R1 on growth performance of weaned piglets prior to LPS challenge.

Items	Day	Control Group	*E. faecium* Group
	0–14	0.23 ± 0.07	0.24 ± 0.07
ADG (Kg/day)	15–21	0.36 ± 0.10	0.38 ± 0.14
	0–21	0.27 ± 0.07	0.29 ± 0.07
	0–14	0.30 ± 0.06	0.33 ± 0.04
ADFI (Kg/day)	15–21	0.63 ± 0.02	0.63 ± 0.04
	0–21	0.41 ± 0.04	0.43 ± 0.03
	0–14	1.41 ± 0.14	1.31 ± 0.10
F/G ratio	15–21	1.78 ± 0.03 ^b^	1.56 ± 0.09 ^a^
	0–21	1.57 ± 0.21	1.41 ± 0.08
Diarrhea rate (%)	0–21	9.16 ± 1.12 ^b^	4.95 ± 0.93 ^a^

Data are means ± SD, *n* = 8 for the control group and *n* = 8 for the *E. faecium* group. ^a,b^ Within a row, means with different superscripts differ (*p* < 0.05).ADG = average daily gain; ADFI = average daily fed intake; F/G = feed/gain ratio. Control group = weaned piglets fed the basal diet; *E. faecium* group = weaned piglets fed the basal diet supplemented with 6.5 × 10^6^ CFU/g *E. faecium* R1.

**Table 4:** Effects of dietary supplementation with *E. faecium* R1 on plasma biochemical parameters of weaned pigs after LPS challenge.

Items	Control Group	LPS Group	*E. faecium* Group
ALT (U/L)	42.8 ± 9.4	44.0 ± 14.8	39.7 ± 14.4
AST (U/L)	41.7 ± 13.2 ^a^	58.8 ± 9.5 ^b^	48.0 ± 8.4 ^a^
TBIL (μmol/L)	4.4 ± 0.8 ^a^	8.2 ± 1.1 ^b^	8.7 ± 1.7 ^b^
TP (g/L)	48.7 ± 3.3	46.7 ± 6.1	46.2 ± 3.7
ALB (g/L)	27.0 ± 4.1	25.6 ± 4.6	26.4 ± 2.0
CHOL (μmol/L)	1.83 ± 0.24	1.84 ± 0.50	1.69 ± 0.38
TG (mmol/L)	0.38 ± 0.13	0.63 ± 0.42	0.65 ± 0.40
BUN (mmol/L)	2.72 ± 0.85	3.39 ± 1.86	2.98 ± 1.08
ALP (U/L)	270.5 ± 53.2	343.3 ± 95.7	304.1 ± 81.0
Crea (μmol/L)	86 ± 9	108 ± 24	100 ± 28
GLU (mmol/L)	5.95 ± 0.94	5.14 ± 1.82	4.39 ± 1.42
CL (mmol/L)	107 ± 2	107 ± 2	108 ± 2
GGT (U/L)	35.4 ± 6.7	45.3 ± 39.4	51.6 ± 25.6

Data are means ± SD, *n* = 8 for the control group, *n* = 8 for the LPS (lipopolysaccharide) group and *n* = 8 for the *E. faecium* group.^a,b^ Within a row, means with different superscripts differ (*p* < 0.05).ALT = alanine aminotransferase; AST = aspartate aminotransferase; TBIL = total bilirubin; TP = total protein; ALB =albumin; CHOL = total cholesterol ;TG = triglyceride; BUN = blood urine nitrogen; ALP = alkaline phosphatase; Crea = creatinine; GLU = glucose; CL = chlorine; GGT = γ-glutamyltransferase.

**Table 5:** Effects of dietary supplementation with *E. faecium* R1 on IL-1β and hormones in plasma and liver of weaned pigs after LPS challenge.

Items	Control Group	LPS Group	*E. faecium* Group
Plasma			
COR (ng/mL)	53.12 ± 16.38 ^a^	140.18 ± 80.30 ^b^	221.20 ± 39.85 ^c^
GC (μIU/mL)	154.09 ± 64.72 ^a^	287.62 ± 150.04 ^a,b^	316.24 ± 195.61 ^b^
INS (μIU/mL)	5.57 ± 2.23	4.64 ± 3.61	7.16 ± 4.65
IGF-1 (ng/mL)	256.85 ± 91.04 ^a^	346.45 ± 47.38 ^b^	304.01 ± 75.11 ^a,b^
PGE2 (pg/mL)	29.31 ± 3.09 ^a^	30.04 ± 2.40 ^a^	30.70 ± 3.19 ^a^
IL-1β (pg/mL)	0.05 ± 0.02	0.06 ± 0.03	0.06 ± 0.03
Liver			
IGF-1 (ng/mg wet weight)	133.79 ± 34.41	156.12 ± 33.28	153.71 ± 25.21
PGE_2_ (pg/mg wet weight)	2.01 ± 0.35 ^b^	1.90 ± 0.27 ^b^	1.47 ± 0.19 ^a^
IL-1β (pg/mg wet weight)	0.29 ± 0.07 ^a^	0.35 ± 0.07 ^a^	0.44 ± 0.10 ^b^

Data are means ± SD, *n* = 8 for the control group, *n* = 8 for the LPS (lipopolysaccharide) group and *n* = 8 for the *E. faecium* group. ^a,b^ Within a row, means with different superscripts differ (*p* < 0.05).COR = Cortisol; GC = glucagon; INS = insulin; IGF-1 = insulin-like growth factor 1; PGE_2_ = prostaglandin (PG) E2; IL-1β = interleukin-1 beta.

**Table 6:** Effects of dietary supplementation with *E. faecium* R1 on the redox status of weaned piglets.

Items	Control Group	LPS Group	*E. faecium* Group
CAT			
Plasma (U/mL)	2.68 ± 1.21	2.87 ± 1.08	2.82 ± 1.50
Pancreas (U/mg protein)	15.01 ± 1.44 ^b^	12.78 ± 1.28 ^a^	17.08 ± 1.72 ^b^
Duodenum (U/mg protein)	1.63 ± 0.51	1.42 ± 0.39	1.26 ± 0.29
Ileum (U/mg protein)	9.52 ± 1.20	9.08 ± 2.93	8.51 ± 2.94
GSH-Px			
Plasma (U/mL)	354.45 ± 53.79	336.94 ± 77.93	332.11 ± 37.45
Pancreas (U/mg protein)	124.32 ± 20.15	124.80 ± 14.56	125.59 ± 19.98
Duodenum (U/mg protein)	29.56 ± 6.17	29.20 ± 5.87	30.02 ± 6.68
Ileum (U/mg protein)	65.46 ± 12.92	69.15 ± 14.03	56.50 ± 9.38
tNOS			
Plasma (U/mL)	20.34 ± 1.45	20.58 ± 5.20	17.30 ± 2.46
Pancreas (U/mg protein)	17.74 ± 1.38 ^a^	17.09 ± 1.90 ^a^	14.91 ± 1.10 ^b^
Duodenum (U/mg protein)	8.32 ± 1.07	7.53 ± 0.66	7.99 ± 2.09
Ileum (U/mg protein)	8.62 ± 2.24	8.35 ± 2.05	7.92 ± 2.00
Colon (U/mg protein)	10.16 ± 2.56	7.90 ± 0.97	9.64 ± 6.02
iNOS			
Plasma (U/mL)	4.90 ± 1.58	4.92 ± 1.48	4.31 ± 2.24
Liver (U/mg protein)	3.19 ± 1.75	2.84 ± 0.93	3.43 ± 1.34
Jejunum (U/mg protein)	4.25 ± 1.53	3.57 ± 1.26	4.97 ± 1.18
Colon (U/mg protein)	3.71 ± 1.40	3.13 ± 0.83	4.65 ± 2.11
MPO			
Plasma (U/mL)	32.78 ± 3.82 ^a^	44.74 ± 3.23 ^b^	38.89 ± 3.45 ^a^
Liver (U/mg protein)	0.33 ± 0.30	0.18 ± 0.04	0.35 ± 0.17
Pancreas (U/mg protein)	0.23 ± 0.08	0.18 ± 0.10	0.19 ± 0.07
Duodenum (U/mg protein)	0.20 ± 0.10	0.23 ± 0.07	0.23 ± 0.08
Jejunum (U/mg protein)	0.21 ± 0.12	0.29 ± 0.09	0.35 ± 0.20
Colon (U/mg protein)	0.21 ± 0.12	0.23 ± 0.13	0.27 ± 0.10
MDA			
Plasma (nmol/mL)	3.75 ± 1.00	3.55 ± 0.42	4.50 ± 1.21
Liver (nmol/mg protein)	8.46 ± 1.40 ^a^	7.81 ± 1.64 ^a^	5.80 ± 1.25 ^b^
Pancreas (nmol/mg protein)	1.26 ± 0.34	1.21 ± 0.57	1.43 ± 0.41
duodenum (nmol/mg protein)	3.33 ± 1.24	3.20 ± 0.82	3.20 ± 1.09
Jejunum (nmol/mg protein)	2.41 ± 0.73	2.21 ± 0.95	2.43 ± 1.54
Ileum (µg/g protein)	2.32 ± 0.37	3.58 ± 1.42	2.56 ± 1.60
Colon (nmol/mg protein)	5.95 ± 2.27	5.44 ± 1.45	3.92 ± 1.83

Data are means ± SD, *n* = 8 for the control group, *n* = 8 for the LPS (lipopolysaccharide) group, and *n* = 8 for the *E. faecium* group. All data are expressed as means ± SD and analyzed by one-way analysis of variance. Differences among treatment means were determined by Duncan’s post hoc test. ^a,b^ Within a row, means with different superscripts differ (*p* < 0.05).CAT = catalase; GSH-Px = glutathione peroxidase; tNOS = total nitric oxide synthase; iNOS = inducible nitric oxide synthase; MPO = myeloperoxidase; MDA = malondialdehyde.

**Table 7:** Effects of dietary supplementation with *E. faecium* R1 on the intestinal digestive and absorption function in weaned piglets.

Items	Control Group	LPS Group	*E. faecium* Group
D-xylose (mmol/L)			
plasma	1.53 ± 0.34 ^b^	0.84 ± 0.60 ^a^	0.79 ± 0.47 ^a^
AKP (U/mg protein)			
Duodenum	71.51 ± 22.20 ^b^	61.39 ± 24.81 ^a,b^	43.92 ± 15.32 ^a^
Jejunum	82.13 ± 37.10 ^b^	49.68 ± 25.09 ^a^	46.41 ± 26.51 ^a^
Ileum	90.68 ± 40.07	70.47 ± 41.94	55.54 ± 20.58
Colon	2.87 ± 1.10	3.72 ± 2.12	3.64 ± 0.93
Maltase (U/mg protein)			
Jejunum	739.87 ± 313.91	588.03 ± 291.51	535.56 ± 281.23
Ileum	1098.62 ± 507.26	790.51 ± 520.02	632.89 ± 446.26
Colon	138.61 ± 35.43	132.24 ± 34.38	113.51 ± 39.03
Lactase (U/mg protein)			
Duodenum	161.86 ± 99.47	144.94 ± 83.05	106.33 ± 64.23
Jejunum	271.86 ± 172.24 ^b^	109.52 ± 55.77 ^a^	159.36 ± 116.13 ^a,b^
Ileum	30.48 ± 12.05	24.73 ± 14.40	18.67 ± 5.75
Colon	36.75 ± 8.78 ^b^	33.45 ± 8.78 ^a,b^	25.13 ± 11.86 ^a^
Sucrase (U/mg protein)			
Duodenum	70.19 ± 42.92	50.30 ± 44.96	34.33 ± 31.51
Jejunum	471.79 ± 290.61 ^b^	175.69 ± 140.78 ^a^	229.16 ± 211.64 ^a^
Ileum	150.25 ± 75.42	117.55 ± 114.16	81.12 ± 64.20

Values are mean ± SD, *n* = 8 for the control group, *n* = 8 for the LPS (lipopolysaccharide) group and *n* = 8 for the *E. faecium* group. ^a,b^ Within a row, means with different superscripts differ (*p* < 0.05).

**Table 8:** Effects of dietary supplementation with *E. faecium* R1 on mRNA expression of *villin*, *Bcl-x1*, and *pBD-1* in Jejunum or Ileum of weaned piglets after LPS challenge.

Items	Control Group	LPS Group	*E. faecium* Group
Villin			
Jejunum	1.000 ± 0.226 ^a^	0.660 ± 0.165 ^b^	1.108 ± 0.230 ^a^
Ileum	1.000 ± 0.160 ^a^	0.630 ± 0.136 ^b^	1.067 ± 0.136 ^a^
Bcl-x1			
Ileum	1.000 ± 0.168 ^b^	0.928 ± 0.195 ^b^	1.601 ± 0.382 ^a^
pBD-1			
Ileum	1.000 ± 0.239 ^b^	1.371 ± 0.356 ^b^	3.079 ± 0.620 ^a^

Data are means ± SD, *n* = 8 for the control group, *n* = 8 for the LPS group, and *n* = 8 for the *E. faecium* group. ^a,b^ Within a row, means with different superscripts differ (*p* < 0.05).

## Data Availability

The data presented in this study are available on request from the corresponding author. The data are not publicly available due to privacy.
